# Faricimab for retinal vein occlusion: a review of current evidence and future perspectives

**DOI:** 10.3389/fphar.2025.1646806

**Published:** 2025-10-14

**Authors:** Yongjie Jing, Daming Li

**Affiliations:** ^1^ Department of Ophthalmology, Dandong First Hospital, Dandong, China; ^2^ Department of Cataract, Dalat Banner Chaoju Eye Hospital, Ordos, China

**Keywords:** faricimab, retinal vein occlusion, vascular endothelial growth factor a, angiopoietin-2, review

## Abstract

Retinal vein occlusion (RVO) is the second most common retinal vascular cause of vision loss after diabetic retinopathy and can lead to substantial visual impairment due to retinal ischemia, hemorrhage, vascular leakage, and macular edema. Intravitreal anti-vascular endothelial growth factor (VEGF) therapy is currently the first-line treatment for RVO-associated macular edema, with its efficacy confirmed by numerous large-scale randomized controlled trials. However, VEGF is not the only pathological driver in RVO. Angiopoietin-2 (Ang-2) has emerged as a key factor that contributes to disease progression by destabilizing the vascular endothelium through disruption of the Tie2 signaling pathway, increasing vascular permeability, and intensifying inflammation. Faricimab is a bispecific monoclonal antibody that targets both VEGF-A and Ang-2, providing a dual mechanism of action that includes anti-permeability, anti-angiogenic, and anti-inflammatory effects. In both the BALATON and COMINO trials, patients with RVO achieved mean best-corrected visual acuity (BCVA) improvements of approximately +17 to +19 letters, along with central subfield thickness (CST) reductions exceeding 300 μm in both treatment arms after Faricimab treatment. Compared to traditional single-target anti-VEGF agents, Faricimab has demonstrated non-inferior or even superior outcomes in visual and anatomical improvements, while also offering extended dosing intervals and reduced treatment burden. Nevertheless, its safety profile positions it mid-range among anti-VEGF therapies. This review outlines the molecular rationale, key clinical trial data, comparative efficacy and safety, and current challenges and future directions for Faricimab in RVO management, aiming to inform its clinical application in RVO and broader retinal vascular disorders.

## Introduction

Retinal vein occlusion (RVO) is the second most common retinal vascular cause of blindness worldwide after diabetic retinopathy (Laouri et al., 2011). Clinically, it is characterized by retinal hemorrhage, capillary leakage, macular edema, and retinal ischemia, often resulting in acute or subacute visual impairment, with severe cases potentially leading to irreversible vision loss (Hamid et al., 2008; Yau et al., 2008). Based on the site and extent of venous involvement, RVO is divided into branch retinal vein occlusion (BRVO) and central retinal vein occlusion (CRVO), both of which can lead to secondary macular edema (ME), which is the primary cause of vision deterioration among these patients. Population-based research has estimated the prevalence of BRVO to range from 0.5% to 2.0%, while CRVO affects approximately 0.1%–0.2% of the population. Over a 15-year period observation, the incidence has been reported at around 1.8% for BRVO and 0.2% for CRVO (Laouri et al., 2011).

Currently, intravitreal injections of anti-vascular endothelial growth factor (anti-VEGF) agents are the standard treatment for RVO-associated ME (Bulla and Lavinsky, 2025). These therapies have been extensively validated in large-scale clinical trials, demonstrating their ability to improve retinal anatomy and visual outcomes (Chen et al., 2025). However, VEGF is not the sole pathogenic factor in the complex pathophysiology of RVO. Recent research has highlighted the pivotal role of angiopoietin-2 (Ang-2), which regulates vascular stability, inflammation, and permeability (Kuriyama et al., 2024). Elevated levels of Ang-2 in RVO may exacerbate vascular leakage and inflammation, thereby contributing to disease progression.

Faricimab (Vabysmo^®^, Genentech/Roche) is a novel bispecific monoclonal antibody that simultaneously targets VEGF-A and Ang-2 within a single molecular framework (Shirley, 2022, Faricimab, 2025, Gene, 2025). By co-inhibiting these two critical signaling pathways, Faricimab aims to achieve more potent and durable anti-permeability and anti-inflammatory effects. It has already demonstrated promising efficacy and extended dosing intervals in the treatment of diabetic macular edema and neovascular age-related macular degeneration, and ongoing clinical trials are expanding its application to other retinal vascular disorders such as RVO (Panos et al., 2023).

This review aims to systematically summarize the current evidence regarding the use of Faricimab in RVO, including its mechanisms of action, efficacy and safety data, and comparative advantages over existing therapies. By integrating findings from both basic research and clinical studies, we hope to provide new insights and potential therapeutic strategies for the management of RVO, ultimately contributing to improved long-term outcomes and patient care.

## Literature search methods

We conducted a narrative review using PubMed, Embase, and Web of Science databases. Keywords included “faricimab”, “retinal vein occlusion”, “BRVO”, “CRVO”, “dual inhibition”, and “Angiopoietin-2”. Articles published from January 2000 to May 2025 were included. Reference lists of relevant studies were manually reviewed. Inclusion criteria comprised English-language clinical trials, basic research, and real-world studies on faricimab in RVO. Reviews, case reports, and non-peer-reviewed sources were excluded.

### Pathophysiological changes in RVO

Retinal vein obstruction causes poor circulation, leading to hypoxia, endothelial damage, and retinal dysfunction (Feng et al., 2025). Endothelial damage increases vascular permeability, causing plasma leakage and leading to macular or retinal edema. (Valeriani et al., 2023). Macular edema can cause significant widespread blurred vision and visual field defects (Bertoli et al., 2021). Hypoxia triggers neovascularization to compensate for poor perfusion, but the new vessels are fragile, prone to leakage and bleeding, worsening edema and vision loss, and potentially leading to vitreous hemorrhage and retinal detachment (Soetikno et al., 2024; Nishimura et al., 2025).

Generally, VEGF is a homodimeric protein that stimulates endothelial cell growth and increases vascular permeability, playing a critical role in angiogenesis and vascular repair (Kuriyama et al., 2024). Additionally, retinal hypoxia caused by thrombosis-induced blood flow obstruction stimulates Ang-2 expression (Inagaki et al., 2021). Ang-2 competitively binds to the Tie2 receptor with Angiopoietin-1 (Ang-1), thereby inhibiting the vascular stabilization effect mediated by Ang-1 through the Tie2 signaling pathway (Heier et al., 2021; Joussen et al., 2021). The elevated Ang-2 level, similar to VEGF, promotes the formation of fragile neovascular structures, exacerbates retinal edema and inflammatory responses (Zhang et al., 2021). Previous study has shown that intraocular Ang-2 levels are elevated in various retinal diseases, including RVO (Regula et al., 2016).

Systemic diseases like hypertension contribute to RVO by disrupting hemodynamics, promoting vasospasm, slowing venous flow, and increasing the risk of thrombosis (Oh et al., 2021). In addition, atherosclerosis (AS), retinal vasculitis, glaucoma, and orbital tumors exerting compression have also been implicated in the pathogenesis of RVO (Jiang et al., 2025; Sen et al., 2025).

## Risk factors for RVO

Numerous studies have demonstrated that both systemic and local ocular factors are closely associated with the development of RVO (Tang et al., 2022; Lendzioszek et al., 2024). Systemic factors include aging, hypertension, diabetes mellitus, hyperlipidemia, and atherosclerosis, which can promote endothelial dysfunction and venous blood flow stasis, thus triggering thrombosis (Kuhli-Hattenbach et al., 2010). Moreover, hypercoagulable states such as hyperhomocysteinemia and antiphospholipid syndrome, along with smoking and obesity as parts of metabolic syndrome, markedly elevate the risk of RVO (Yau et al., 2008).

Regarding ocular factors, glaucoma and elevated intraocular pressure increase retinal venous resistance and lead to venous pressure elevation and blood flow stagnation, serving as major local triggers for RVO (Călugăru and Călugăru, 2021). BRVO is mainly caused by mechanical compression, while CRVO relates to hypercoagulability and inflammation; both lead to ischemia, leakage, and inflammation (Ip and Hendrick, 2018). These processes further induce the overexpression of VEGF and Ang-2, promoting macular edema formation and increased vascular permeability.

Although conventional anti-VEGF therapy has achieved favorable results in controlling RVO-associated macular edema, it still leaves a therapeutic gap in addressing Ang-2–mediated vascular instability and chronic inflammation. Therefore, dual-pathway targeted therapies against both VEGF-A and Ang-2 have garnered increasing attention.

## Overview of faricimab

Faricimab is an innovative bispecific antibody that simultaneously inhibits VEGF-A and Ang-2. It entered clinical trials in 2017 and was approved by the U.S. Food and Drug Administration (FDA) in January 2022 for the treatment of neovascular age-related macular degeneration (nAMD) and diabetic macular edema (DME). In September of the same year, the European Union also approved Faricimab for the same indications. In 2024, China approved the clinical use of Faricimab. At present, Faricimab has been approved in over 40 countries and regions worldwide, and is widely used in the treatment of various retinal vascular diseases, including nAMD, DME, and RVO (Khanani et al., 2021; Nichani et al., 2024).

Clinically, Faricimab is administered via intravitreal injection, like other anti-VEGF drugs. Since Faricimab is administered directly into the eye, it exerts its therapeutic effects locally, thereby reducing the risk of systemic exposure. This mode of administration enhances the local efficacy of the drug while minimizing potential systemic adverse reactions. In the ocular metabolic process, Faricimab is degraded by lysosomal pathways similar to endogenous IgG into small peptides and amino acids, with a systemic half-life of approximately 7.5 days (Abdeldaim and Schindowski, 2023).

During the initial treatment phase, the injection frequency of Faricimab can be adjusted to every 4, 8, 12, or 16 weeks based on the patient’s anatomical response and visual improvement until optimal visual or anatomical outcomes are achieved (Glassman et al., 2020; Ciulla et al., 2021). Studies have shown that more than 60% of patients can extend the injection interval to once every 4 months after completing this periodic treatment, while still maintaining and improving visual acuity (Teo et al., 2023). The development of this innovative drug not only signifies a breakthrough in the field of ophthalmic treatment but also offers patients a more effective and longer-lasting therapeutic option, demonstrating significant clinical potential and application prospects.

### Mechanisms of faricimab in the treatment of RVO

#### Faricimab inhibits the Ang-2 pathway

**FIGURE 1:**
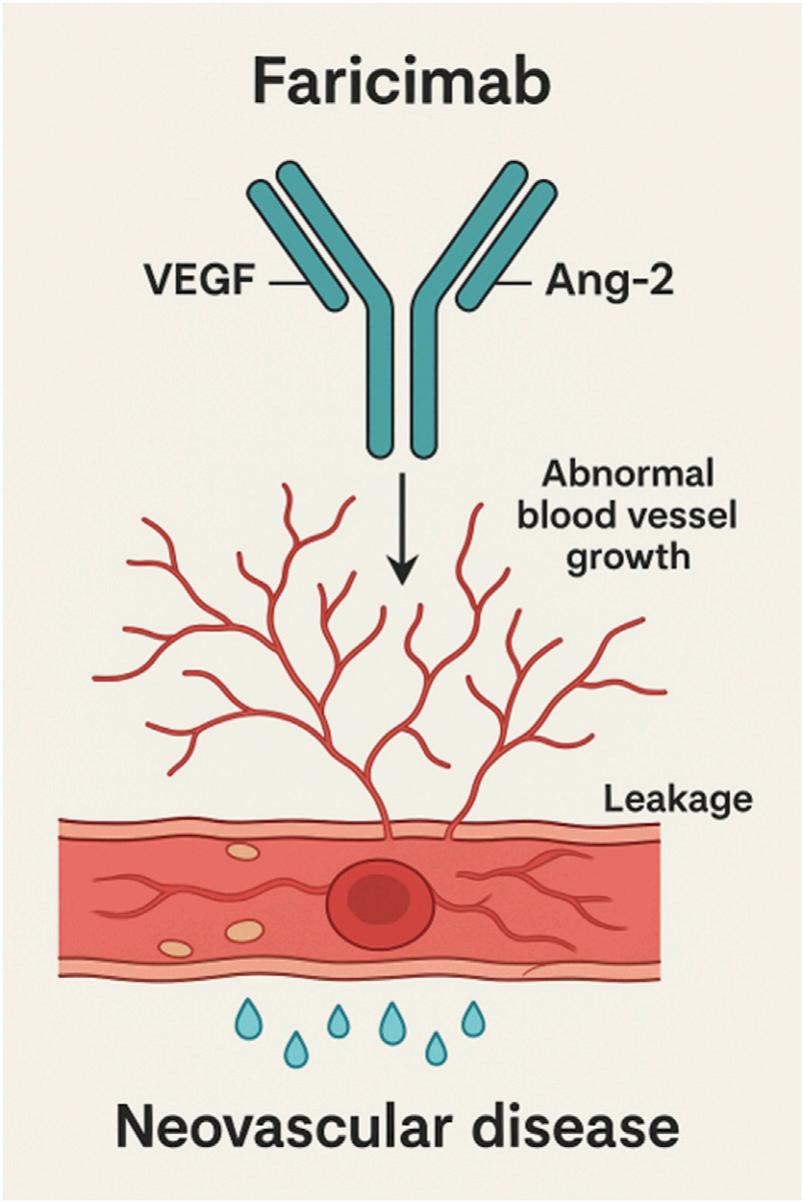
Molecular mechanism of action of faricimab leading to a decrease of vascular leakage and the inhibition of neovascular processes.

Faricimab exerts its therapeutic effect by directly binding to Ang-2, thereby preventing its interaction with the Tie2 receptor and suppressing Ang-2-induced vascular instability (Heier et al., 2021). This effectively reduces pathological neovascularization and increased vascular permeability caused by Ang-2 overexpression. By inhibiting Ang-2 activity, Faricimab restores the normal interaction between Ang-1 and Tie2 receptor, thereby promoting vascular stability and alleviating inflammatory responses. This mechanism is critical for reducing retinal edema and improving vision, particularly in retinal diseases driven by increased vascular permeability and neovascularization (Panos et al., 2023). Faricimab’s mechanism of action was shown in Figure 1.

#### Dual inhibitory mechanism of faricimab

In RVO, vascular endothelial growth factor-A (VEGF-A) binds to VEGF receptor-2 (VEGFR-2), promoting endothelial cell proliferation, migration, and neovascularization. Overexpression of VEGF-A leads to abnormal neovascularization and accumulation of retinal fluid, resulting in macular edema and vision loss. Faricimab binds to VEGF-A, blocking its interaction with VEGFR-2, thereby inhibiting VEGF-A-mediated signaling pathways. This effectively reduces endothelial cell proliferation, migration, and tube formation, demonstrating significant efficacy in controlling disease progression and improving visual outcomes (Nicolò et al., 2021; Ferro Desideri et al., 2022). In addition to VEGF-A inhibition, Faricimab also targets Ang-2, which, under pathological conditions, disrupts vascular stability and enhances the pro-angiogenic effects of VEGF, thereby aggravating retinal damage (Hussain et al., 2019). By simultaneously inhibiting both VEGF-A and Ang-2 pathways, Faricimab synergistically attenuates VEGF-driven angiogenesis, reduces inflammation, and enhances vascular barrier function (Liberski et al., 2022; Shirley, 2022). The bispecific targeting mechanism of Faricimab offers unique advantages in controlling RVO, particularly in suppressing inflammatory responses, maintaining vascular stability, and comprehensively improving the retinal microenvironment, thereby providing prolonged vascular stability and reducing the risk of disease recurrence (Khanani et al., 2021; Nicolò et al., 2021).

### Comparison of faricimab and traditional therapies in RVO treatment

#### Faricimab vs. Anti-VEGF agents

Anti-VEGF agents such as bevacizumab, ranibizumab, and aflibercept are currently the mainstay for treating macular edema secondary to RVO. These drugs neutralize VEGF-A and block its interaction with VEGF receptors, thereby suppressing VEGF-mediated angiogenesis and increased vascular permeability (Shughoury et al., 2023). Although intravitreal anti-VEGF therapy is effective, it also carries risks, including intraocular infection, vitreous hemorrhage, and retinal detachment.

Previous study has shown that Faricimab provides a safety profile comparable to other anti-VEGF agents in patients with RVO, but it offers a distinct advantage in treatment durability, particularly in patients with refractory retinal diseases (Literature Commentary, 2023). Faricimab targets both VEGF-A and Ang-2, thereby more effectively stabilizing vascular structures and prolonging drug efficacy. According to a clinical study by Tadayoni et al. (2024), patients treated with Faricimab showed faster improvements in best corrected visual acuity (BCVA) during early treatment, with sustained benefits up to week 24. Additionally, the Faricimab group demonstrated significant reductions in central retinal thickness (CRT), greater anatomical improvement, lower rates of macular leakage, and good drug tolerability.

A meta-analysis revealed that Faricimab could reduce the annual number of injections compared to other anti-VEGF therapies (Mean Difference = −2.42, 95% Confidence Interval, CI [-3.93 to −0.90], p = 0.002), while still achieving comparable visual improvements (Li et al., 2023). This reduced injection frequency not only lowers the risk of ocular and systemic complications associated with intravitreal injections but also improves patient compliance. A recent meta-analysis showed that while all anti-VEGF agents (aflibercept 2 mg, bevacizumab, faricimab, and ranibizumab) are effective for RVO-induced macular edema, faricimab demonstrated the highest improvement in BCVA, with a pooled result of 16.9 (95% CI: 8.794 to 25.006, p < 0.001), reflecting the best efficacy in the study (Chen et al., 2025). Of note, head-to-head RVO data beyond week 24 are still limited.

However, it is also worth noting that faricimab carries a higher risk of adverse effects compared to bevacizumab and ranibizumab, although still lower than aflibercept (Chen et al., 2025). Therefore, clinical decisions should comprehensively consider both efficacy and safety. Moreover, a study from San Francisco, California, reported that higher cost-effectiveness of Faricimab over other agents (Ishida et al., 2023). However, these results may not be generalizable to health systems in Europe or Asia due to variation in drug pricing, reimbursement policies, and healthcare infrastructure.

#### Faricimab vs. glucocorticoids

Intravitreal glucocorticoid (GC) injections are an effective treatment for RVO, particularly in patients who are unresponsive to or contraindicated for anti-VEGF therapies. GCs exert potent anti-inflammatory and anti-edematous effects by reducing the release of inflammatory mediators, inhibiting VEGF expression, decreasing vascular permeability, and stabilizing the blood-retinal barrier, thereby alleviating retinal edema and improving vision (van der Wijk et al., 2019; Ryu et al., 2024). Common GCs include triamcinolone acetonide (TA), dexamethasone implant (Ozurdex), and fluocinolone acetonide intravitreal implant (Iluvien). These drugs also inhibit neovascularization and help prevent RVO complications.

Currently, no direct head-to-head studies comparing Faricimab and GCs in RVO treatment are available. However, a study by Covello et al. (2024) demonstrated that dexamethasone treatment significantly improved retinal structure disorganization, external membrane damage, macular ischemia, CRT, and BRVO symptoms, indicating that both therapies are effective in relieving RVO symptoms. Nevertheless, the effect of GCs is often most pronounced after the first injection and may diminish with repeated administration. In contrast, Faricimab, by inhibiting both VEGF-A and Ang-2 pathways, provides longer-lasting effects and may extend dosing intervals to 3–4 months.

In terms of indications, Faricimab is primarily used for macular edema caused by RVO, whereas GCs are more often used in inflammatory conditions or DME. For different RVO subtypes, Faricimab may be more effective in complex CRVO, while both therapies offer comparable efficacy in reducing macular edema in BRVO. However, adverse events such as intraocular pressure elevation and cataracts are more commonly associated with GCs. Therefore, clinicians should tailor treatment choices to individual patient conditions and needs.

### Clinical studies of faricimab in the treatment of RVO

#### Preclinical studies

Preclinical studies further confirmed the critical role of Ang-2 in retinal diseases, particularly in the process of cytokine-induced vascular leakage. Faricimab demonstrated the ability to simultaneously inhibit VEGF-A and Ang-2, effectively protecting human vascular endothelial cells from damage (Wong et al., 2025). In the spontaneous choroidal neovascularization (CNV) JR5558 mouse model, Faricimab significantly reduced vascular permeability and neovascular lesions, as well as retinal edema, neuronal apoptosis, and macrophage infiltration by simultaneously inhibiting both VEGF and Ang-2, compared to inhibition of either factor alone (Canonica et al., 2023). Another study showed that intravitreal injection of Faricimab had a more pronounced effect in reducing leaky lesions and effectively alleviated laser-induced CNV formation (Toma et al., 2010; Zhang et al., 2020).

#### Clinical studies

BALATON and COMINO are two global multicenter Phase III clinical trials aimed at comprehensively validating the efficacy and safety of Faricimab in the treatment of RVO (Tadayoni et al., 2024). BALATON evaluated the efficacy and safety of Faricimab in treating macular edema secondary to BRVO, while COMINO focused on CRVO-related macular edema (Hattenbach et al., 2023). These two trials enrolled a total of 1,282 patients, including 553 in the BALATON trial and 729 in the COMINO trial. Participants were randomly assigned to receive either Faricimab 6.0 mg or Aflibercept 2.0 mg, with injections every 4 weeks for a total of six doses. The trial results showed that after receiving Faricimab every 4 weeks for up to 24 weeks, patients in the Faricimab group experienced more significant visual improvement. In contrast, faricimab was associated with a higher percentage of patients achieving the absence of macular leakage. This suggests that faricimab’s dual mechanism of action, which involves inhibition of both VEGF and angiopoietin-2, may offer benefits beyond VEGF inhibition alone, addressing aspects of retinal vascular pathology more thoroughly and improving anatomical outcomes in patients with RVO. Another study also confirmed that Faricimab not only regulates neovascular permeability but also significantly reduces macular edema, improves visual acuity, and enhances overall ocular health (Aljundi et al., 2024). Although current studies on the use of Faricimab in RVO are still limited, by analyzing treatment outcomes in diseases with similar pathophysiology, such as DME and nAMD, it has been observed that Faricimab significantly improves BCVA, reduces central subfoveal thickness (CST), and improves both anatomical and functional parameters, while showing good tolerability and safety throughout the treatment course (Raimondi et al., 2024; Toto et al., 2024). These clinical findings further support the potential advantages of Faricimab in the treatment of RVO and provide important clinical evidence for its application in retinal vascular diseases.

While the BALATON and COMINO trials demonstrated favorable efficacy and safety profiles of Faricimab in controlled clinical settings, real-world data from neovascular age-related macular degeneration (nAMD) cohorts have raised concerns regarding adverse events such as intraocular inflammation and retinal vasculitis (Cozzi et al., 2024; Liu et al., 2025). These discrepancies may be attributed to differences in patient populations, monitoring rigor, and treatment adherence between tightly controlled trials and routine clinical practice. Additionally, faricimab’s dual inhibition mechanism targeting both VEGF-A and Ang-2 might contribute to distinct immunologic responses not fully captured in earlier studies.

Moreover, the current evidence predominantly covers outcomes up to week 24, leaving the long-term safety and durability of Faricimab therapy less defined. This limitation highlights the need for extended follow-up to monitor potential delayed adverse effects and to better understand how sustained dual-pathway inhibition influences retinal health over time. The optimal retreatment regimen, balancing efficacy, safety, and treatment burden, remains to be established. It is crucial to determine individualized dosing intervals, as patient responses can vary widely, and overtreatment or undertreatment may impact visual outcomes and safety. Ongoing and future large-scale, long-term studies are therefore essential to provide comprehensive insight into Faricimab’s risk-benefit profile, refine personalized treatment protocols, and confirm its role in routine clinical practice beyond initial trials.

Generally, Ranibizumab, aflibercept, and Faricimab are the primary anti-VEGF agents used in the treatment of RVO, each with distinct molecular targets and clinical profiles. Ranibizumab and aflibercept primarily inhibit VEGF-A, though aflibercept also binds VEGF-B and placental growth factor (PlGF), while faricimab uniquely targets both VEGF-A and Ang-2, enabling dual-pathway inhibition. All three agents demonstrated robust visual acuity gains of approximately 17–19 letters by week 24 in their respective pivotal trials—BRAVO and CRUISE for ranibizumab (Varma et al., 2012), COPERNICUS and GALILEO for aflibercept (Heier et al., 2014; Korobelnik et al., 2014), and BALATON and COMINO for Faricimab. Central subfield thickness (CST) reductions were similarly significant across agents, typically ranging from 300 to over 450 μm.

In terms of dosing, ranibizumab and aflibercept are administered monthly (Q4W), though aflibercept may be extended to Q8W in practice. Faricimab offers greater flexibility, with individualized extension intervals up to every 16 weeks (Q16W); by week 68, over half of patients in BALATON and COMINO achieved ≥ Q12W dosing. All three agents exhibited favorable safety profiles in clinical trials; however, post-marketing real-world data have raised concerns about intraocular inflammation and vasculitis with faricimab, underscoring the importance of ongoing long-term surveillance.

The Canadian Drug Expert Committee (CDEC) raised concerns regarding the reliability of the economic analysis, emphasizing that the incremental quality-adjusted life-year (QALY) gain projected for faricimab over other anti-VEGF agents was not substantiated by the indirect evidence provided. The committee acknowledged methodological limitations of the indirect comparison, including potential heterogeneity and wide credible intervals, yet noted that the available data generally indicate little or no meaningful difference in BCVA outcomes between faricimab and other anti-VEGFs administered on flexible dosing regimens. Consequently, the extent to which faricimab may deliver superior health outcomes and QALY benefits in patients with RVO remains uncertain (Faricimab (Vabysmo): Indication: For the treatment of macular edema secondary to retinal vein occlusion: Reimbursement Recommendation, 2025).

Several novel approaches are under investigation for RVO, including gene therapies, and long-acting delivery systems. Gene therapies and sustained-expression platforms show promise for reducing injection frequency, though concerns about immunogenicity, dose control, and irreversibility remain (Lendzioszek et al., 2024). The Port delivery system (PDS) is a refillable, non-biodegradable implant that enables continuous delivery of drug to the vitreous. As of September 2024, the PDS is currently being evaluated in Phase III trials for DME, as well as diabetic retinopathy without DME, however there are no current trials underway for RVO(Genentech. Pipeline. September 2024. Available at: https://www.gene.com/medical-professionals/pipeline). Compared with these modalities, Faricimab provides a clinically validated, titratable dual-target therapy with established efficacy, although head-to-head studies will be required to determine its position relative to emerging options.

### Prospects

As the first bispecific antibody that simultaneously inhibits VEGF-A and Ang-2, Faricimab reduces vascular leakage and neovascularization, showing potential advantages over traditional anti-VEGF therapies. Its dual mechanism brings new hope and possibilities for the treatment of retinal vascular diseases such as RVO. In the BALATON and COMINO clinical trials, Faricimab demonstrated visual improvement effects comparable to those of Aflibercept (Chang et al., 2024; Tadayoni et al., 2024). Furthermore, Faricimab’s longer dosing interval observed in other indications significantly reduced treatment frequency and outpatient visits, which is particularly important for patients who require long-term injection therapy, effectively alleviating the healthcare burden and improving patient compliance (Mansour et al., 2020). The unique mechanism of Faricimab provides a valuable supplement to traditional treatment approaches for RVO and may potentially change the treatment landscape for many patients in the future.

Looking ahead, although Faricimab has shown remarkable clinical potential in the treatment of RVO, current clinical data and experimental studies on its application in RVO remain relatively limited. Future research should focus on large-scale, randomized controlled clinical trials to more comprehensively assess the long-term efficacy and safety of Faricimab in treating RVO-related macular edema among different ethnic or cultural populations. In China, Faricimab has only been approved for use in BRVO patients, which reflects the need to evaluate its efficacy in different subtypes of RVO (e.g., CRVO and BRVO). Additionally, future research directions for Faricimab may expand to other ophthalmic diseases related to vascular abnormalities, such as pathological myopia and retinopathy of prematurity (ROP). Particular attention should also be given to investigating the feasibility and effectiveness of Faricimab in combination with traditional anti-VEGF drugs, GC, and other treatment modalities for RVO, especially in cases where monotherapy is insufficient or where patients exhibit complex pathological characteristics. Combination therapy may significantly enhance treatment efficacy, reduce recurrence, and prolong treatment intervals. Moreover, future research should address the economic and social benefits of Faricimab, and through appropriate medical policies and support measures, further improve its accessibility. With continuous advancement in related studies, the outlook for Faricimab in ophthalmic treatment will become increasingly promising, potentially driving more precise and personalized management of RVO.

Future studies should prospectively evaluate biomarkers that predict response to Faricimab. Candidate biomarkers include aqueous or vitreous concentrations of VEGF-A and Ang-2, inflammatory cytokine panels (IL-6, IL-8), and imaging biomarkers such as OCT/OCTA-derived metrics (CST, foveal avascular zone size, deep capillary density) and microperimetry. Multi-omic approaches (proteomics/metabolomics) and machine-learning models that integrate imaging and molecular data could enable enrichment designs that enroll patients most likely to obtain durable benefit from dual VEGF/Ang-2 inhibition.
